# Creating and Validating a Questionnaire for Assessing Dentists’ Self-Perception on Oral Healthcare Management—A Pilot Study

**DOI:** 10.3390/healthcare12090933

**Published:** 2024-05-01

**Authors:** Silviu Catalin Tibeica, Elena Raluca Baciu, Iulian Costin Lupu, Carina Balcos, Ionut Luchian, Dana Gabriela Budala, Andreea Tibeica, Zinovia Surlari, Elena Mihaela Carausu

**Affiliations:** 1 Department of Health Management, Faculty of Dental Medicine, “Grigore T. Popa” University of Medicine and Pharmacy, 16 Universității Street, 700115 Iași, Romaniaelena.carausu@umfiasi.ro (E.M.C.); 2 Department of Dental Materials, Faculty of Dental Medicine, “Grigore T. Popa” University of Medicine and Pharmacy, 16 Universității Street, 700115 Iași, Romania; 3 Department of Oral Health, Faculty of Dental Medicine, “Grigore T. Popa” University of Medicine and Pharmacy, 700115 Iași, Romania; 4 Department of Periodontology, Faculty of Dental Medicine, “Grigore T. Popa” University of Medicine and Pharmacy, 16 Universității Street, 700115 Iași, Romania; 5 Department of Dentures, Faculty of Dental Medicine, “Grigore T. Popa” University of Medicine and Pharmacy, 700115 Iaşi, Romania; dana-gabriela.bosinceanu@umfiasi.ro; 6 Department of Implantology, Faculty of Dental Medicine, “Grigore T. Popa” University of Medicine and Pharmacy, 16 Universității Street, 700115 Iași, Romania; 7 Department of Fixed Dentures, Faculty of Dental Medicine, “Grigore T. Popa” University of Medicine and Pharmacy, 16 Universității Street, 700115 Iași, Romania

**Keywords:** dental management, quality of life, patient satisfaction, quality management, dental services, dentistry

## Abstract

Background and Objectives: Questionnaires designed to test knowledge and self-perception can be valuable tools for diagnosing a dentist’s understanding of the management and administration of a practice. The objective of this study was to create and authenticate a questionnaire for assessing dentists’ self-perception on oral healthcare management developed from discussions with experts in this field. Material and Methods: In order to create and verify a questionnaire survey, a cross-sectional, descriptive, and analytical study was carried out. Participants’ personal information and 31 statements across four categories made up the final questionnaire form. The answers to the questionnaire were in the form of a Likert scale. After refining the initial version, a total of 36 interviews were conducted at dental offices to verify the validity. For the Exploratory Factor Analysis (EFA), we used the Kaiser–Meyer–Olkin (KMO) index, the Bartlett sphericity test, and also Cronbach alpha coefficient for the validity of the questionnaire. Results: The accuracy of the instrument was measured by intrarater and interrater reliability. For the EFA, all the communalities exceeded the threshold of 0.05. With a Cronbach’s alpha coefficient of 0.898, the questionnaire has sufficient internal consistency. Conclusions: The questionnaire demonstrates robust reliability and validity, thereby affirming its suitability for its intended purpose.

## 1. Introduction

The importance of efficient management in dental practices cannot be overstated. As healthcare providers, dentists are tasked with offering high-quality care while also navigating the complexities of running a successful business [1]. In order for a managerial organization to be successful, it is essential to have clinicians who possess significant clinical knowledge. Without this expertise, even a well-run organization is destined to fail [2].

This dual responsibility requires a comprehensive management approach that balances patient care with operational efficiency [3]. Professionals possessing extensive dental management expertise are more capable of adjusting to these changes, implementing inventive solutions, and assuming leadership roles [4,5].

The interaction between the users’ and the professionals’ behavior inside this system is what drives health service utilization, which in turn drives healthcare operations [6]. There are several surveys available in the scientific literature that focus on dental management. These surveys try to evaluate many elements, including patient satisfaction, quality of care, and the impact of oral health on overall quality of life [7,8]. They offer vital information about the viewpoints of patients, the performance of clinicians, and the effectiveness of the healthcare system [9,10,11].

The issues covered encompass a broad spectrum, such as treatment results, pain control, healthcare accessibility, and patient inclinations. Questionnaires are essential instruments for analyzing and improving the delivery of dental care and the experience of patients; among them, we can find Dental Treatment Satisfaction Questionnaire [6], Dental Anxiety Scale (DAS) [12], and Dental Fear Survey (DFS) [13]. These questionnaires provide standardized tools for gathering data, evaluating patient perspectives, and improving the delivery of dental care.

Through the use of validated questionnaires, dental professionals and researchers can collect standardized data, enhance patient communication, and enhance decision-making processes in dental care [14].

The evolving landscape of dental healthcare, marked by technological advancements and changing patient expectations, further necessitates dynamic management practices [15].

Dental offices have their own special mix of possibilities and threats in the dynamic healthcare system. Managing a practice efficiently and profitably while simultaneously providing excellent patient care calls for a high level of expertise [16].

Patient relationships, financial stability, personnel dynamics, technology progress, regulatory compliance, and clinical quality are all part of this complex duty. An integrated management plan that is in line with the overall objectives of providing quality care and sustainable business practices is necessary due to the intricacy of these elements [17,18].

A number of factors are causing changes in the oral healthcare sector. These include financial investors, who have opened numerous large dental centers or chains, an increase in the number of dentists employed, the possibility of an oversupply in urban areas and an undersupply in rural areas, and a shortage or migration of skilled personnel [19,20,21]. Change is also brought about by new areas of study like AI and big data, as well as by the technical and professional difficulties that come with research advancements [22,23,24]. Everyone involved in dental healthcare, from patients to dentists, might be impacted by these issues [25,26].

The objective of this study was to create and validate a Dental Management Survey developed from discussions with experts in this field, with the aim of assessing dentists’ perceptions and practices related to clinical and office management.

## 2. Materials and Methods

### 2.1. Research Design

In order to create and verify a questionnaire survey, a cross-sectional, descriptive, and analytical study was carried out. The creation and validation of the questionnaire followed a three-stage process. After a thorough literature review on marketing and management was conducted, an expert in the field of dentistry marketing and management examined the content validity of the suggested instrument.

In order to test and maybe reformulate the instrument, the first version of the questionnaire was distributed to ten managers of clinics or individual offices in N-E of Romania. Afterward, the responses were analyzed, and the questions were modified to make them as easy to understand as possible.

### 2.2. Participants

The next step was to send the survey, which was hosted on the Survio platform—https://www.survio.com/ro/, accessed on 8 January 2024, to clinics and office managers via email in order to recruit a sample for the survey’s final validation. The questionnaire was answered by 36 managers of clinics or practices, a number considered sufficient for conducting the pilot study.

Reliability holds significant importance throughout various scientific areas. When the measurements of the outcome are expressed in numerical values, the reliability can be assessed by calculating the intraclass correlation coefficient (ICC). When strategizing a dependability study, it is crucial to ascertain the minimal number of participants. Having an excessive number of participants can be time-consuming and may also lead to an increase in the research expenditure. On the other hand, having too few individuals can negatively affect the accuracy of the ICC estimate, making it impossible to draw any conclusions from the study [27,28,29].

The test-retest reliability will be assessed using intra-class correlations (ICC), with a minimum acceptable reliability (ρ0) of 0.85 and a desired reliability (ρ1) of 0.9. Based on these factors, the sample size was determined to be 16 dentists for each edition of the questionnaire. Given a dropout rate of 10%, at least 18 dentists were calculated. We doubled the number calculated, and we determined a sample of 36 dentists.

✓ Inclusion criteria
  ❖ Doctors who answered the whole questionnaire;
  Private practice owners.

✓ Exclusion criteria
  ❖ Doctors who have not responded to all questions and have left aspects incomplete.

All participants gave their consent after being informed, and any personal information was taken out from the data to protect their privacy. Informed consent was obtained from all participants, and personal identifiers were removed from the data to ensure anonymity. The study received approval from the Institutional Review Board of UMF Gr.T.Popa Iasi-Nr. 318/30.05.2023.

### 2.3. Questionnaire Structure

Participants’ personal information (gender, age, specialization, years of practice, dental office location, education, current professional status, kind of practice, and practice type) and 31 statements across 4 categories made up the final questionnaire form.

There are 4 domains: Location domain, which includes references to the office’s layout and design (3 statements); Patient domain, which deals with patient care (7 statements), Management domain, which covers the day-to-day operations of the clinic or office (13 statements), and medical, which deals with the relationship between management and Medic (8 statements). The answers to the questionnaire were in the form of a Likert scale: yes, no, I don’t know [30]. The Likert scale was translated into measurable scores by assigning numerical values to each response option.

### 2.4. Statistical Analyses

To check the rehabilitation and validity of the questionnaire, we used SPSS 26.0 0 (IBM SPSS Statistics for Windows, version 26.0, IBM Corp., Armonk, NY, USA). The statistical analysis included frequencies, mean values, and differences evaluated by means of chi-square. To evaluate the validity of the questionnaire, we determined the values of the Cronbach alpha coefficient and the correlation coefficients (Cronbach-alpha coefficient > 0.70) [31].

The internal consistency of a questionnaire, or its domain(s), can be measured using Cronbach’s alpha, which is used for the reliability assessment of questionnaires. Typically, a Likert scale or other interval-based metric is used to assess the response variable. The dependability of a psychometric instrument was the first purpose of the test’s development by Cronbach [31]. The items are measuring the same latent variable or dimension if Cronbach’s alpha, which runs from 0 to 1, is higher. The inverse is true when Cronbach’s alpha is small, close to zero; this indicates that the items in the questionnaire do not measure the same dimension, rendering the questionnaire unreliable and inconsistent.

For the Exploratory Factor Analysis (EFA), we used the Kaiser–Meyer–Olkin (KMO) index, which shows us the correct sample size (KMO must have a value > 0.50/0.65) [29].

KMO is determined by looking at how the variables are correlated with each other. It can take on values between 0 and 1, with higher values indicating that the variables are correlated and that factor analysis would work well with the data and lower values indicating that the variables are uncorrelated and that there could not be a common factor impacting them [32].

We also used the Bartlett sphericity test, which tests the hypothesis that the correlation matrix inter-items (R) is different from a unity matrix (*p* < 0.05). Finally, varimax rotation was used to see the final selection of items. Statistical significance was established for *p* = 0.05 [33].

## 3. Results

A total of 36 participants who provided complete responses to the questionnaire were chosen to participate in the validation research. With a range of 32 to 59 years, the study group had an average age of 41.44 ± 7.85 years, with 52.8% of the subjects being male. Over half of the people who took part are general dentists who have no particular training. Sixty-seven percent of the participants are dentists with less than ten years of experience, whereas twenty-five percent have more than twenty years of experience. Among all participants, 75% are dentists who also work as practice managers, and 58.3% have a single dental office as their organizational structure (Table 1).

With a Cronbach’s alpha coefficient of 0.898, the questionnaire has sufficient internal consistency. With a range of 0.700 to 0.885, the average Cronbach alpha coefficient for the “Location domain” was 0.866. The “Patient domain” has a Cronbach alpha coefficient of 0.981 on average (range: 0.976 to 0.982). The average Cronbach alpha coefficient for the “Management domain” was 0.975 (min 0.971 and max 0.977), while for the “Medical domain” it was 0.910 (min 0.899 and max 0.943), as shown in Table 2.

The Exploratory Factor Analysis (EFA) utilized principal component analysis and varimax rotation. The lowest factor loading criterion was established at 0.05. The communality of the scale is evaluated to determine the extent to which it explains the variance in each dimension, aiming for a satisfactory level of explanation. Upon examining the table findings, it is evident that all communalities exceeded the threshold of 0.05. The minimum value of 0.474 was observed for item Ma14, while the maximum values of 0.965 were recorded for items P8 and P9. Out of all the communalities, only one has a value that is less than 0.5. However, this value does not have any impact on the overall value of item Ma14, as shown in Table 3.

The suitability of the correlation matrix for factor analysis was indicated by the statistically significant results (x2(n = 36) = 324.574, *p* < 0.001) obtained from evaluating its overall significance using Bartlett’s Test of Sphericity, which provides a measure of the statistical probability that the correlation matrix has a significant correlation with other components. All of the data are suitable for factor analysis, as shown in Table 4, since the Kaiser–Meyer–Olkin measure of sample adequacy was 0.834, which is good (higher than 0.500).

Four scale variables were identified as a result of this research, explaining 83.229% of the observed variance. They started out with Eigenvalues higher than 1. Of the total variation, 39.642% is explained by the first factor. 20.789% of the total variation was explained by the second component, while 17.512% and 5.286% of the total variance were determined by the third and fourth variables, respectively (Table 5).

**Figure 1:**
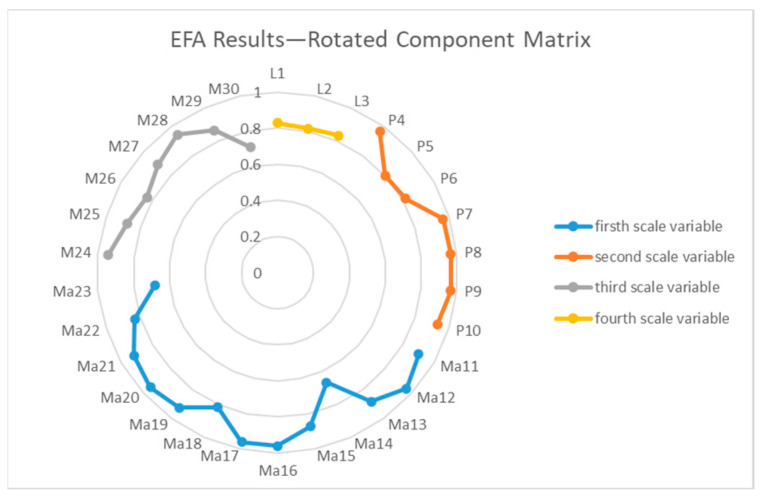
EFA Results—Rotated Component Matrix.

After applying a rotation transformation to increase interpretability, a rotated component matrix in factor analysis illustrates the link between the observable variables and the underlying factors. In our study, all items were imported correctly into the reference domain, meaning they accurately reflect the data from their respective domains. However, we had to eliminate one item, M31, because this item did not correctly fit into the domain it was part of and run the EFA analysis again because of it (Figure 1).

This EFA alignment included the identification of four components. Factor 2 compiles items from P4 to P10, which represent the Patient domain, while Factor 1 includes items from Ma11 to Ma23, which pertain to the Management domain. Items M24–M30 make up Factor 3, which stands for the Physician field, while items L1–L3 make up Factor 4, which stands for the Location field. This arrangement of questions shows that each field was filled out correctly and that the questionnaire may be used for its intended purpose.

## 4. Discussion

Dentists were included in the sample after they were conveniently contacted to take part in the study by digital means. In comparison to more traditional methods, such as mail, digital media have many advantages. These include being more efficient, reaching more people in more places, costing less, and providing just as good of a response. As a result, digital media have been increasingly popular in recent years.

Critical parts of evaluating psychometric scales, according to the American Psychological Association, are descriptive measures of reliability and construct validity. All of these factors were thoroughly examined in this research.

The results indicate robust reliability and validity of the questionnaire, with a Cronbach’s alpha coefficient of 0.898, suggesting sufficient internal consistency. The factors identified through EFA explain 83.229% of the observed variance, divided into four main domains: clinic location, patient care, daily management, and the relationship between management and doctors. Thus, the form of the questionnaire is considered appropriate for the intended purpose, highlighting its promise as an effective tool for self-perception of dental practitioners regarding the management of clinical and office activities. These results fall within the range of values that have been accepted in previous studies [34,35].

Validating a measurement instrument among the Romanian population assists in identifying the perceived importance among physicians of proper clinic management. This aids in assessing the level of knowledge regarding dental management phenomena and their manifestations, as well as understanding how physicians utilize their personal coping resources functionally when patient referrals decrease [36].

The utility and purpose of this study are integrated into an expanded model of public health management and play a significant role in providing individual and group interventions and programs at the county and national levels concerning proper and beneficial dental clinic management methods.

In the ever-evolving field of dentistry, the significance of dental management knowledge cannot be overstated. As dental practices strive to provide exceptional patient care, the integration of effective management practices becomes pivotal [29]. This encompasses not only the clinical proficiency of dental professionals but also their ability to adeptly navigate administrative duties, patient relations, and practice sustainability. Understanding dental management principles is essential for ensuring the smooth operation of dental clinics, enhancing patient satisfaction, and ultimately contributing to the overall success of the practice [37].

Moreover, the advent of new technologies, changing patient expectations, and the increasing complexity of healthcare regulations add layers of complexity to dental practice management. Professionals equipped with robust dental management knowledge are better positioned to adapt to these changes, implement innovative solutions, and lead their practices toward sustainable growth [21,38]. Furthermore, alongside the introduction of new machinery, there have also been recent advancements in compounds for oral care. These compounds have been shown to have a substantial impact on the oral environment. Future research and questionnaires should investigate the potential of postbiotics, probiotics [39], or bacterial pieces such as lysates [40] to affect clinical and microbiological parameters in dental patients.

The practical significance of this study has been realized. Gaining insights into perceptions regarding a factor with the potential to significantly impact professional activity, along with understanding the methods and pathways for influencing these perceptions, may pave the way for identifying effective means of control.

A few limitations should be taken into consideration in interpreting the results of this study. First, the subjects were not randomly selected, although random selection would have been preferable because it was based on volunteerism. Also, it is possible that some responses may have been socially desirable, meaning that subjects may have intuited the true purpose of the experiment. Although administering the questionnaires did not pose difficulties, their interpretation must be performed with caution, as the instrument captured only a declarative level of participants.

A possible recommendation for future research is to consider these limitations and control for them to reduce the likelihood of confounding variables appearing.

The study also can have possible biases like social desirability, response, Hawthorne, confirmation, and sampling biases, which may impact the questionnaire’s validity. When talking about social desirability, participants may provide responses that they believe are socially desirable rather than reflecting their true perceptions and behaviors. This could occur if dentists feel pressure to respond in a certain way due to the nature of the study or the presence of the researcher.

Hawthorne effect refers to participants who may alter their behavior or responses simply because they are aware that they are being observed or studied. This could influence the results of the pilot study and affect the validity of the questionnaire.

In order to mitigate biases, we ensured random participant selection, minimized social pressure, used standardized procedures, and maintained participant anonymity.

This paper contributes to the existing literature by providing a validated tool that can be used to enhance the understanding and application of effective management practices in the dental field, a crucial aspect for success in oral health care. By addressing aspects such as clinic design, patient satisfaction, daily operations, and medical collaboration, the questionnaire offers a comprehensive perspective on the key factors that influence the quality of management in dental practices.

## 5. Conclusions

The questionnaire demonstrates robust reliability and validity, thereby affirming its suitability for its intended purpose. This survey instrument holds promise as an effective instrument of dentists’ self-perception concerning the management of clinical and dental office activities.

## Figures and Tables

**Table 1:** Demographic characteristics of the study group.

Demographics	No	%
Age	41.44 ± 7.85 years (min. 32–59 years)	
Gender		
male	17	47.2
female	19	52.8
Specialty		
no specialty	20	55.6
specialist	16	44.4
Years of practice		
0–10 years	24	66.7
11–20 years	3	8.3
21–30 years	9	25.0
Dental office location		
Urban	28	77.8
Rural	8	22.2
Studies		
University studies	31	86.1
Postgraduate studies	5	13.9
Current professional status		
Doctor and manager	27	75.0
Manager	9	25.0
Type of practice		
Individual dentistry office	21	58.3
Dental clinic	15	41.7

**Table 2:** Mean value, SD, Cronbach alfa coefficient, and Correlation coefficient for all items.

		Mean Value	SD	Cronbach Alfa	Correlation Coefficient	Cronbach Alfa Mean Value Domain
Domain location						
1.	I consider the aesthetic aspect of the cabinet as acceptable for the patients who frequent it.	1.67	0.632	0.700	0.794 **	0.866
2.	I believe that patients can easily reach the office.	1.56	0.607	0.830	0.711 **	
3.	I consider that certain functional and aesthetic elements of the office need improvement.	1.86	0.593	0.885	0.538 **	
Domain patient						
4.	The patients are satisfied with the medical team’s performance.	1.44	0.652	0.976	0.890 **	0.981
5.	Digital communication is the best method for maintaining the office.	1.56	0.735	0.982	0.782 **	
6.	Offering discounts on dental treatments represents the best method for retaining patients.	1.56	0.735	0.981	0.841 **	
7.	Ensuring punctuality within the medical team is crucial for keeping patients.	1.44	0.695	0.977	0.939 **	
8.	I offer all my patients equal care and attention, regardless of their status.	1.42	0.649	0.976	0.967 **	
9.	The patients are satisfied with the types of treatments we offer.	1.42	0.649	0.976	0.967 **	
10.	Patients frequently complete satisfaction questionnaires regarding the doctors’ performance and the quality of services provided.	1.53	0.696	0.977	0.916 **	
Domain management						
11.	I have selected the medical team to provide the best treatment conditions.	1.50	0.655	0.973	0.754 **	975
12.	I have chosen modern medical equipment to be able to offer any type of dental treatment to my patients.	1.44	0.652	0.971	0.870 **	
13.	I am constantly thinking about new methods to attract new patients.	1.47	0.654	0.973	0.767 **	
14.	I believe I have good knowledge of management and marketing.	1.72	0.779	0.979	0.560 **	
15.	I continually strive to stimulate competitiveness among the doctors to increase the clinic’s productivity.	1.50	0.655	0.973	0.733 **	
16.	I believe that proper organization of data in the office facilitates its functioning.	1.39	0.645	0.971	0.880 **	
17.	I constantly need to verify the existing stock of materials in the office, as well as the functioning of the equipment.	1.39	0.645	0.971	0.880 **	
18.	For the optimal functioning of the clinic, it is essential to have strategies and targets that need to be achieved constantly.	1.44	0.695	0.974	0.754 **	
19.	A good manager needs to have knowledge and negotiation skills.	1.42	0.649	0.972	0.840 **	
20.	“The satisfied patients bring in more patients”—represents the primary method through which the number of patients grows in the current practice.	1.42	0.649	0.971	0.840 **	
21.	I consider the patient scheduling system that I use to be efficient.	1.44	0.695	0.972	0.880 **	
22.	I consider the use of software for activity management to be essential for proper clinic management.	1.56	0.735	0.974	0.713 **	
23.	I participated in management and marketing courses.	1.83	0.561	0.977	0.545 **	
Domain doctor						
24.	Open communication with the medical team members must be continuously encouraged.	1.28	0.566	0.900	0.408 *	0.910
25.	I consider the work of medical and auxiliary staff to be of high quality, as they are well-prepared.	1.36	0.639	0.909	0.820 **	
26.	Continuously, I pay attention to the comments and suggestions of my colleagues.	1.33	0.632	0.909	0.771 **	
27.	I encourage and support colleagues to pursue professional training courses.	1.36	0.639	0.899	0.820 **	
28.	I believe that the medical team must continuously update their knowledge in the field to enhance performance.	1.28	0.566	0.900	0.911 **	
29.	The activity of my colleagues is supported by the quality of the devices and materials available in the cabinet.	1.36	0.639	0.904	0.820 **	
30.	The doctors who collaborate in my clinic are constantly informed about the latest developments in their field of expertise.	1.50	0.737	0.920	0.616 **	
31.	Some collaborating doctors frequently change their workplace.	1.97	0.696	0.943	0.310 **	

** Correlation is significant at the 0.01 level (2-tailed). * Correlation is significant at the 0.05 level (2-tailed).

**Table 3:** Communalities that reveal the degree of variation in each domain.

	Communalities	Extraction
L1	I consider the aesthetic aspect of the cabinet as acceptable for the patients who frequent it	0.881
L2	I believe that patients can easily reach the office	0.787
L3	I consider that certain functional and aesthetic elements of the office need improvement.	0.769
P4	The patients are satisfied with the medical team’s performance.	0.949
P5	Digital communication is the best method for maintaining the office.	0.899
P6	Offering discounts on dental treatments represents the best method for retaining patients.	0.916
P7	Ensuring punctuality within the medical team is crucial for keeping patients.	0.946
P8	I offer all my patients equal care and attention, regardless of their status.	0.965
P9	The patients are satisfied with the types of treatments we offer.	0.965
P10	Patients frequently complete satisfaction questionnaires regarding the doctors’ performance and the quality of services provided.	0.907
Ma11	I have selected the medical team to provide the best treatment conditions.	0.825
Ma12	I have chosen modern medical equipment to be able to offer any type of dental treatment to my patients.	0.945
Ma13	I am constantly thinking about new methods to attract new patients.	0.832
Ma14	I believe I have good knowledge of management and marketing.	0.474
Ma15	I continually strive to stimulate competitiveness among the doctors to increase the clinic’s productivity.	0.790
Ma16	I believe that proper organization of data in the office facilitates its functioning.	0.962
Ma17	I constantly need to verify the existing stock of materials in the office, as well as the functioning of the equipment.	0.962
Ma18	For the optimal functioning of the clinic, it is essential to have strategies and targets that need to be achieved constantly.	0.762
Ma19	A good manager needs to have knowledge and negotiation skills.	0.889
Ma20	“The satisfied patients bring in more patients”—represents the primary method through which the number of patients grows in the current practice.	0.926
Ma21	I consider the patient scheduling system that I use to be efficient.	0.867
Ma22	I consider the use of software for activity management to be essential for proper clinic management.	0.706
Ma23	I participated in management and marketing courses	0.552
M24	Open communication with the medical team members must be continuously encouraged.	0.902
M25	I consider the work of medical and auxiliary staff to be of high quality, as they are well-prepared.	0.787
M26	Continuously, I pay attention to the comments and suggestions of my colleagues.	0.736
M27	I encourage and support colleagues to pursue professional training courses.	0.826
M28	I believe that the medical team must continuously update their knowledge in the field to enhance performance.	0.916
M29	The activity of my colleagues is supported by the quality of the devices and materials available in the cabinet.	0.784
M30	The doctors who collaborate in my clinic are constantly informed about the latest developments in their field of expertise.	0.538
M31	Some collaborating doctors frequently change their workplace.	0.832

**Table 4:** Kaiser–Meyer–Olkin and Bartlett’s Test.

Kaiser–Meyer–Olkin Measure of Sampling Adequacy		0.834
Bartlett’s Test of Sphericity	Approx. Chi-Square	324.574
	df	55
	Sig.	0.000

**Table 5:** Total Variance Explained.

Component	Initial Eigenvalues			Extraction Sums of Squared Loadings		
	Total	% of Variance	Cumulative %	Total	% of Variance	Cumulative %
1	11.893	39.642	39.642	11.893	39.642	39.642
2	6.237	20.789	60.431	6.237	20.789	60.431
3	5.254	17.512	77.943	5.254	17.512	77.943
4	1.586	5.286	83.229	1.586	5.286	83.229

## Data Availability

All data are available from corresponding authors upon reasonable request.
